# Synthesis and Characterization of Positively Charged Pentacationic [60]Fullerene Monoadducts for Antimicrobial Photodynamic Inactivation

**DOI:** 10.3390/molecules17055225

**Published:** 2012-05-07

**Authors:** Sammaiah Thota, Min Wang, Seaho Jeon, Satyanarayana Maragani, Michael R. Hamblin, Long Y. Chiang

**Affiliations:** 1 Department of Chemistry, Institute of Nanoscience and Engineering Technology, University of Massachusetts, Lowell, MA 01854, USA; 2 Wellman Center for Photomedicine, Massachusetts General Hospital, Boston, MA 02114, USA; 3 Department of Dermatology, Harvard Medical School, Boston, MA 02115, USA; 4 Harvard-MIT Division of Health Sciences and Technology, Cambridge, MA 02139, USA; 5 Department of Laboratory Medicine and Pathobiology, University of Toronto, Toronto, ON M5S 1A8, Canada

**Keywords:** pentacationic C_60_ monoadducts, decacationic C_60_ monoadduct, *N,N′,N,N,N,N*-hexapropyl-hexa(aminoethyl)amine, photosensitizer

## Abstract

We designed and synthesized two analogous pentacationic [60]fullerenyl monoadducts, C_60_(>ME_1_N_6_^+^C_3_) (**1**) and C_60_(>ME_3_N_6_^+^C_3_) (**2**), with variation of the methoxyethyleneglycol length. Each of these derivatives bears a well-defined number of cationic charges aimed to enhance and control their ability to target pathogenic Gram-positive and Gram-negative bacterial cells for allowing photodynamic inactivation. The synthesis was achieved by the use of a common synthon of pentacationic *N,N′,N,N,N,N*-hexapropyl-hexa(aminoethyl)amine arm (C_3_N_6_^+^) having six attached propyl groups, instead of methyl or ethyl groups, to provide a well-balanced hydrophobicity–hydrophilicity character to pentacationic precursor intermediates and better compatibility with the highly hydrophobic C_60_ cage moiety. We demonstrated two plausible synthetic routes for the preparation of **1** and **2** with the product characterization via various spectroscopic methods.

## 1. Introduction

Broad-spectrum one-photon based photodynamic therapy (1*γ*-PDT)-mediated killing of pathogenic Gram-positive (e.g., *Staphylococcus aureus*) and Gram-negative (e.g., *Escherichia coli*) bacterial targets using a conventionally accessible light source is an emerging medical approach to treat infectious diseases, especially, those caused by multi-antibiotic-resistant bacteria [1,2,3,4,5]. The efficacy of 1*γ*-PDT depends on several parameters, including photophysical characteristics of the photosensitizer, the ability of the photosensitizer to target bacteria, the method of administration, and the availability of an appropriate light source. Fullerenes are highly photostable molecules suitable for single-dose multiple-treatments applications. Nearly quantitative efficiency of intersystem crossing from the excited singlet state of [60]fullerene (^1^C_60_*) to its triplet excited state (^3^C_60_*) readily allows intermolecular triplet energy transfer from ^3^C_60_* to molecular oxygen leading to the production of singlet oxygen (^1^O_2_) [6,7], which is highly reactive toward biological substrates producing subsequent cell damage. This photochemical mechanism serves as the basis of photodynamic cytotoxicity against pathogenic microorganisms, including multi-antibiotic-resistant bacteria. However, chemical functionalization of C_60_ is necessary to enhance its solubility in water. In general, attachment of multiple hydroxyl, carboxylic acid, and glycolic oxide addend groups may serve the purpose. Water-solubility of these derivatives increases as the number of hydrophilic groups increases, whether these functional groups are located in either the same addend group or different addend moieties. The latter case leads to the synthesis of fullerenyl multiadducts that may change significantly the molecular orbital configuration of the fullerene cage and, thus, its HOMO–LUMO energy gap level and effectiveness in the production of ^1^O_2_. The 1*γ*-PDT efficiency can be optimized by performing only a limited number of addition reactions to the fullerenyl olefinic bonds to preserve the low HOMO–LUMO energy gap level of the cage. This restriction suggests that [60]fullerene monoadducts using hydrophilic or amphiphilic groups would be suitable candidates. However, since only one addend group is able to be attached to the cage in a monoadducts, sufficient hydrophilicity is required to allow compatibility of the resulting derivative with water. 

It has been demonstrated that polycationic photosensitizers exhibited high activity as 1*γ*-PDT agents for targeting and photokilling against both Gram-positive and Gram-negative bacterial species [8,9]. These reported findings revealed the importance of cell surface interactions between multicationic drug molecules and anionic peptide residues in the cell wall. Specifically, several factors including differences in physiology, cell wall, and cytoplasmic membrane structures between Gram-positive and Gram-negative bacteria [10,11,12] affect the properties of particular functional groups to be attached on the fullerene cage to allow effective targeting selectivity, drug-delivery, and photodynamic inactivation. 

**Scheme 1:**
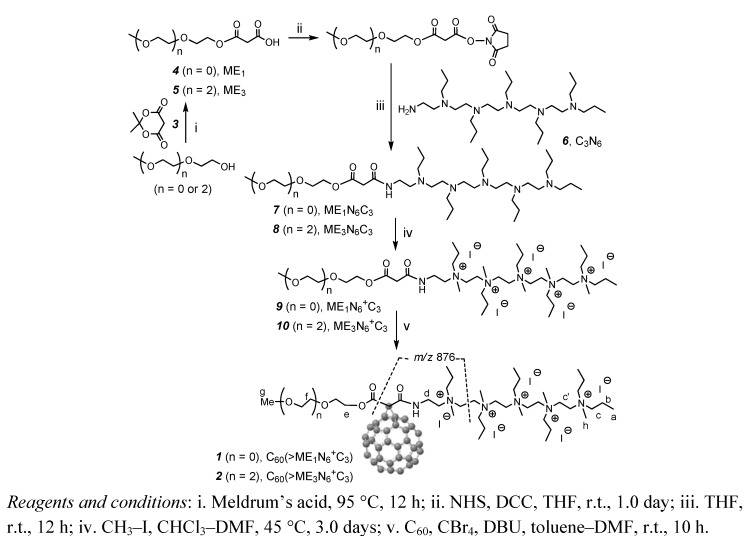
The first synthetic steps of C_60_(>ME_1_N_6_^+^C_3_) **1** and C_60_(>ME_3_N_6_^+^C_3_) **2**.

**Scheme 2:**
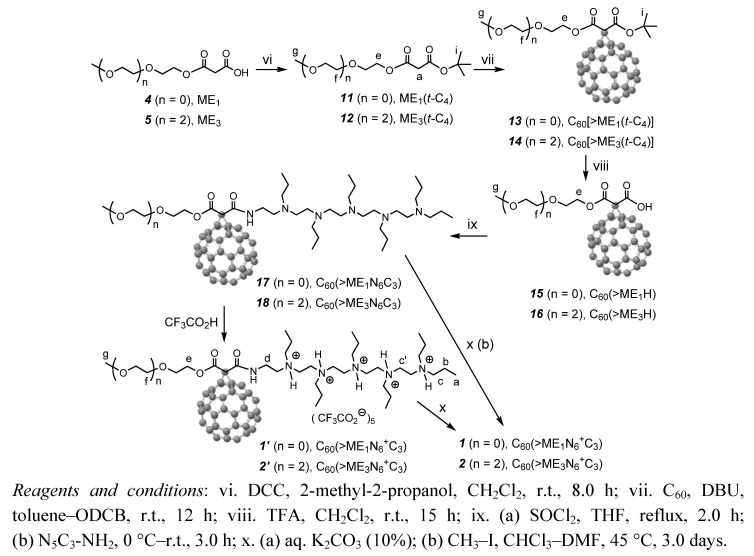
The second synthetic steps of C_60_(>ME_1_N_6_^+^C_3_) **1** and C_60_(>ME_3_N_6_^+^C_3_) **2**.

Accordingly, we considered the structural modification of C_60_ to allow the increase of its solubility in physiologic media and its compatibility in an environment of bacterial disease in tissue. This led to our design and synthesis of [60]fullerenyl monoadducts bearing a well-defined high number of cationic charges that hitherto had remained challenging and has rarely been reported to date. In this paper, we describe a rational linkage of water-soluble quaternary alkylammonium multi-salts and ester-amide functional groups to a well-defined pentacationic arm together with an efficient synthetic method for its attachment on a C_60_ nanocage. The synthesis led to the preparation of new pentacationic [60]fullerene-based nano-photosensitizers **1** and **2**, as shown in Scheme 1 and Scheme 2. 

In these structures, arm moieties each bearing a high number of cationic charges and an amide moiety are capable of inducing the H-bonding in the vicinity of the fullerene cage. 

## 2. Results and Discussion

Enhancement of the hydrophilicity of fullerene derivatives can be achieved by incorporation of the oligo(ethylene glycol) unit [13,14,15], an aminoacid moiety [16], or ionic functional groups [17,18,19] as addend attachments of C_60_ cage. The resulting amphiphilic derivatives have been reported to undergo different forms of solid aggregation in aqueous solution if the hydrophilic moiety of the addend is insufficiently large to overcome the high hydrophobicity of the fullerene cage. To circumvent this solid aggregation problem, we undertook the effort to synthesize a well-defined water-soluble pentacationic *N,N′,N,N,N,N*-hexapropyl-hexa(aminoethyl)amine arm moiety C_3_N_6_^+^, as a charged C_3_N_6_ (**6**), with the number of charge being fixed at five per arm and used as the common synthon in the preparation of [60]fullerene monoadducts, as shown in Scheme 1. One example was given by the combination of a water-compatible ethylene glycol unit with a C_3_N_6_^+^ arm to a single addend, such as the arm precursors ME_1_N_6_^+^C_3_ (**9**) and ME_3_N_6_^+^C_3_ (**10**), to enhance the water-solubility. Synthesis of **9** and **10** began with the reaction of either 2-methoxyethanol or triethylene glycol monomethyl ether with 2,2-dimethyl-1,3-dioxane-4,6-dione (**3**, Meldrum’s acid) at 90–95 °C for a period of 12 h to afford malonic acid methoxyethyleneglycol ester, ME_1_ (**4**), or malonic acid methoxytriethyleneglycol ester, ME_3_ (**5**), in 95 or 90% yield, respectively. Amidation reaction of **4** and **5** was carried out by the treatment with *N*-hydroxysuccinamide and *N*,*N*′-dicyclohexyl carbodiimide (DCC) in anyhydrous THF at ambient temperature over a period of 12 h, followed by the removal of insoluble byproduct of *N,N′-dicyclohexyl* urea and the further treatment with *N,N′,N,N,N,N-*hexapropyl-hexa(aminoethyl)amine, C_3_N_6_ (**6**), for an additional period of 12 h. These reactions resulted in the corresponding products of methoxyethyleneglycol-[*N,N′,N,N,N,N*-hexapropyl-hexa(aminoethyl)-amino]malonamide ester, ME_1_N_6_C_3_ (**7**), and methoxy-tri(ethyleneglycol)-[*N,N′,N,N,N,N*-hexapropyl-hexa(aminoethyl)amino]malonamide ester, ME_3_N_6_C_3_ (**8**), in 82 and 80% yield, respectively. Quaternization reaction of mono- and tri(ethoxylated) hexaaminomalonamide precursors **7** and **8** using methyl iodide as the methylation agent at 45–50 °C for a period of 3.0 days afforded the corresponding methoxyethyleneglycol-[*N,N′,N,N,N,N-*hexapropyl-hexa(aminoethyl)amino]malonamide ester methyl quaternary ammonium salt, ME_1_N_6_^+^C_3_ (**9**), and its tri(ethyleneglycolated) analogue ME_3_N_6_^+^C_3_ (**10**) in 94 and 92% yield, respectively. Attachment of a C_60_ cage on the quaternary ammonium salts of malonamide was accomplished by the treatment of **9** and **10** in DMF with predissolved C_60_ in toluene in the presence of 1.8-diazabicyclo[5.4.0]-undec-7-ene (DBU) at ambient temperature for a period of 8.0 h. In this reaction, carbon tetrabromide was applied as the bromination agent for the replacement of malonyl *α*-proton *in situ*. To minimize the possible formation of partial fullerenyl byproducts containing multiaddends, an excess amount (2.5 equiv.) of C_60_ was applied. At the end of fullerenation, an excessive amount of C_60_ molecules was recovered and removed by repeatedly washing the crude products with toluene until the observation of a clear toluene solution in washings. The reaction procedure led to the isolation of methoxyethyleneglycol-(20-oxo-4,7,10,13,16-pentapropyl-4,7,10,13,16,19-hexaaza-nonadecan-19-yl)[60]fullerenyl malonate quaternary methyl ammonium salt, C_60_(>ME_1_N_6_^+^C_3_) (**1**), and its tri(ethyleneglycolated) analogue C_60_(>ME_3_N_6_^+^C_3_) (**2**) in 55 and 50% yield, respectively.

**Figure 1:**
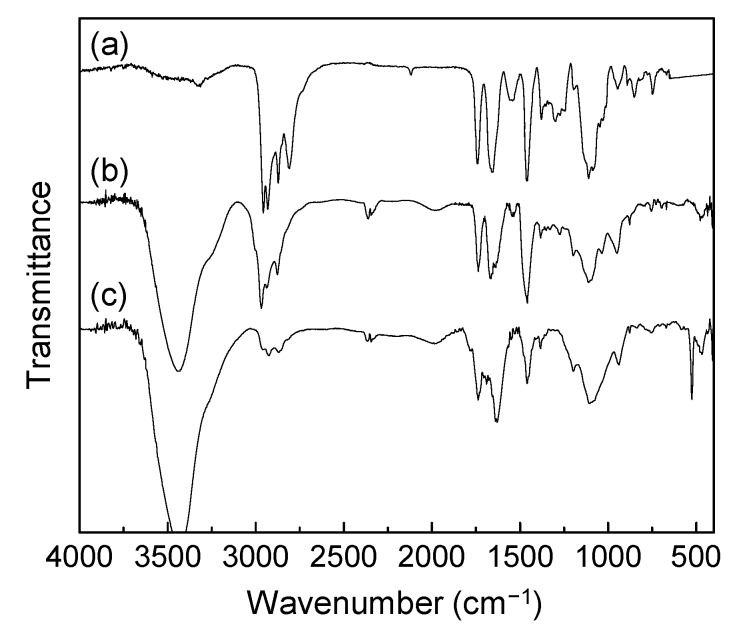
FT-IR spectra of (a) ME_3_N_6_C_3_ **8**, (b) ME_3_N_6_^+^C_3_ **10**, and (c) C_60_(>ME_3_N_6_^+^C_3_) **2**.

Alternatively, as shown in Scheme 2, (ethyleneglycolated)malonic acids **4** and **5** were converted to their corresponding *tert-*butyl(2-methoxyethyl)malonate ester, ME_1_(*t*-C_4_) (**11**), and its tri(ethyleneglycolated) analogue ME_3_(*t*-C_4_) (**12**) in 74 and 77% yield, respectively, byan esterification reaction with *t*-butyl alcohol in anyhydrous dichloromethane (DCM) in the presence of *N,N'-*dicyclohexylcarbodiimide (DCC) at ambient temperature for a period of 8.0 h. Subsequent fullerenyl cyclopropanation reaction of **11** and **12** was carried out by treatment with [60]fullerene solution in a mixture of toluene and 1,2-dichlorobenzene in the presence of carbon tetrabromide and 1.8-diazabicyclo[5.4.0]-undec-7-ene (DBU) over a period of 12 h at room temperature. The products *tert*-butyl(2-methoxyethyl)[60]fullerenyl malonate, C_60_[>ME_1_(*t*-C_4_)] (**13**), and *tert*-butyl(methoxy-triethyleneglycol)[60]fullerenyl malonate, C_60_[>ME_3_(*t*-C_4_)] (**14**), were purified by column chromatography [SiO_2_, toluene−ethyl acetate (20:1) as eluent] as brown solids in 78 and 75% yield, respectively. Direct transamidation reaction of **13** and **14** with C_3_N_6_ was not successful owing to the complicated side-reaction arising from the attack of C_3_N_6_ on C_60_ itself at elevated temperatures which is required for the effective transamidation. Therefore, we carried out the transformation of **13** and **14** to the corresponding methoxyethyleneglycol-(20-oxo-4,7,10,13,16-pentapropyl-4,7,10,13,16,19-hexaaza-nonadecan-19-yl)[60]fullerenyl malonate **17**, C_60_(>ME_1_N_6_C_3_), and its methoxytriethyleneglycol ester analogous **18**, C_60_(>ME_3_N_6_C_3_), respectively, by the acid hydrolysis of **13** and **14** first to 2-[60]fullerenyl-3-(2-methoxyethoxy)-3-oxopropanoic acid **15**, C_60_(>ME_1_H), and its methoxy-triethyleneglycol ester analogous **16**, C_60_(>ME_3_H), respectively. The latter products were obtained as brown solids in yields of 89% and 87%, respectively. Conversion of **15** and **16** to their acid chloride intermediates was carried out by the reaction with thionyl chloride in THF. To minimize the amination of C_3_N_6_ on C_60_, subsequent reaction with the acid chloride intermediate was performed at 0 °C to room temperature over a period of 3.0 h. A relatively pure C_60_(>ME_1_N_6_C_3_) **17** was obtained in 47% yield. It was accompanied with a small quantity of byproduct as a decarboxylated fullerene malonate-monoadduct that is removable. The stability of **17** and **18** in either solution or solid phase was found to be low owing to possible intermolecular complex formation between the hexaamine moiety and C_60_ cage giving insoluble aggregate solids. Therefore, compound **17** and **18** was quaternized to the protonated ammonium–trifluoroacetate salt C_60_(>ME_1_N_6_^+^C_3_) (**1′**) and C_60_(>ME_3_N_6_^+^C_3_) (**2′**), respectively, with trifluoroacetic acid in a short period of time immediately after the workup procedure and purification. Final synthesis of the products pentacationic methoxyethyleneglycol-(20-oxo-4,7,10,13,16-pentapropyl-4,7,10,13,16,19-hexaaza-nonadecan-19-yl)[60]fullerenyl malonate quaternary methyl ammonium salt C_60_(>ME_1_N_6_^+^C_3_) (**1**), and its analogous methoxy-triethyleneglycol ester C_60_(>ME_3_N_6_^+^C_3_) (**2**), was carried out by the neutralization of **1′** and **2′** first with aqueous potassium carbonate in CHCl_3_ as a biphase solution. Resulting [60]fullerenyl malonates **17 **and **18**, respectively, were then transferred to a solvent mixture of anhydrous CHCl_3_ and DMF (2:1) for methyl quaternization with an excess amount of iodomethane added portionwise over a period of 3.0 days at 45 °C to afford **1** and **2**. Successful amidation conversion from malonic acid **5** to ME_3_N_6_C_3_ was verified by the FT***-***IR spectrum of **8** (Figure 1a) showing two clear and strong vibrational carbonyl absorption bands centered at 1667 and 1736 cm^−1^ corresponding to malonylamide carbonyl [–NH-C(=O)–] and ester carbonyl (–C=O) moieties, respectively. In the same spectrum, IR peaks located at 2952, 2928, 2862, and 2805 cm^−1^ were assigned to the stretching absorption bands of aliphatic –C-H. Anti-symmetric deformations of –CH_3_ groups and scissor vibrations of –CH_2_ groups appeared as medium intensity bands centered around 1456 cm^−1^, while symmetric deformations of CH_3_ groups exhibited the absorption around 1379 cm^−1^ that overlaps with the –N-C− stretching vibration of amide [–N-(C=O)–] bands at 1400 cm^−1^. A strong broad band centered at 1104 cm^−1^ was assigned to the stretching vibrations of –C-O-C– and –C-N– moieties. Methyl quaternization of **8**, having tertiary penta(ethylamino) arms, to ME_3_N_6_^+^C_3_**10** with iodide salts showed retention of some of IR absorption bands (Figure 1b), except the shift of –NH-C(=O)– band to 1669 cm^−1^ and a new broad band at 947 cm^−1^, corresponding to the absorption of a number of quaternary –C-N^+^– bonds. Formation of ME_3_N_6_^+^C_3_-attached C_60_monoadduct was verified by the observation of a sharp characteristic fullerene half-cage absorption band of C_60_(>ME_3_N_6_^+^C_3_) **2 **at 524 cm^−1^ in the spectrum of Figure 1c. These results provided a good agreement of the product structure of **2**. Similar infrared absorption bands of C_60_(>ME_1_N_6_^+^C_3_) **1** were observed, except a relatively smaller –C-O-C– band than that of **2** centered at 1000–1200 cm^−1^.

Mass spectroscopic data collection of both **1** and **2** was proven to be difficult due to their polycationic nature and facile fragmentations occurring at the conjunction of the C_60_ cage and the pentacationic malonate arm, giving mainly the highly detectable C_60_ ion mass fragment at *m/z* 721, as displayed in MALDI−TOF mass spectra using sinapic acid as the matrix. All fragmented high mass ions were very weak in intensity including peaks at *m/z* 1930 (M^+^–I^−^) (supporting information) and 2019 (MH^+^–I^−^) as the molecular ion of **1** and **2**, respectively. The most pronounced mass fragmentation ions at *m/z* 874 and 876 in both spectra of **1** and **2** were assigned to the substructure of C_60_[>H(C=O)NHCH_2_CH_2_N^+^-PrMe_2_], indicating a main malonylamide moiety being attached on a C_60_ cage, consistent with the [60]fullerenyl monoadduct structure. 

**Figure 2:**
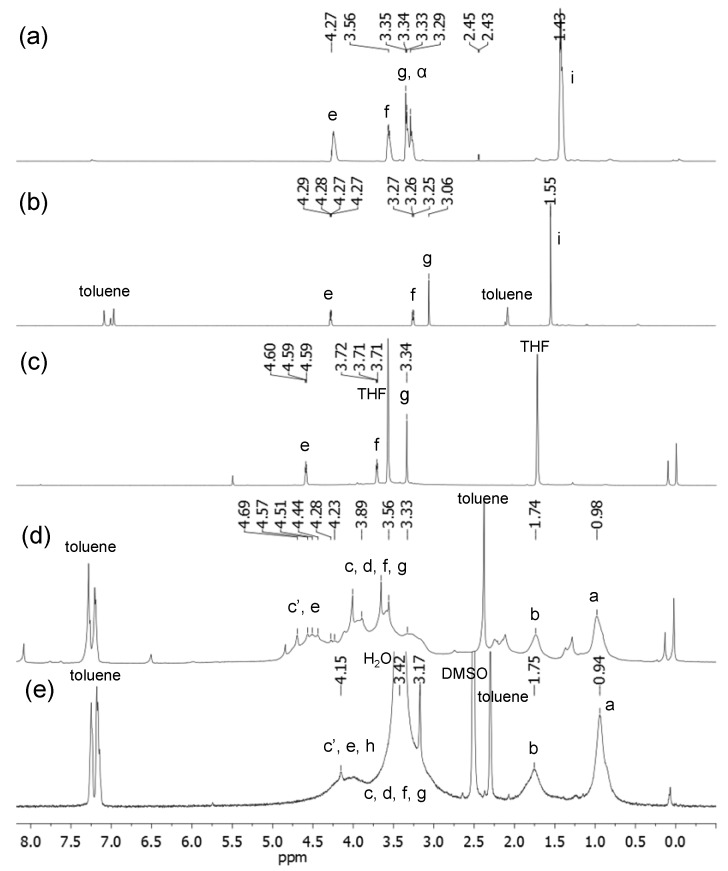
^1^H NMR spectra of (**a**) **11** in CDCl_3_, (**b**) **13** in toluene-*d*_8_, (**c**) **15** in THF-*d*_8_, (**d**) **1’** in CDCl_3_−toluene-*d*_8_, and (**e**) **1** in DMSO-*d*_6_–toluene-*d*_8_.

**Figure 3:**
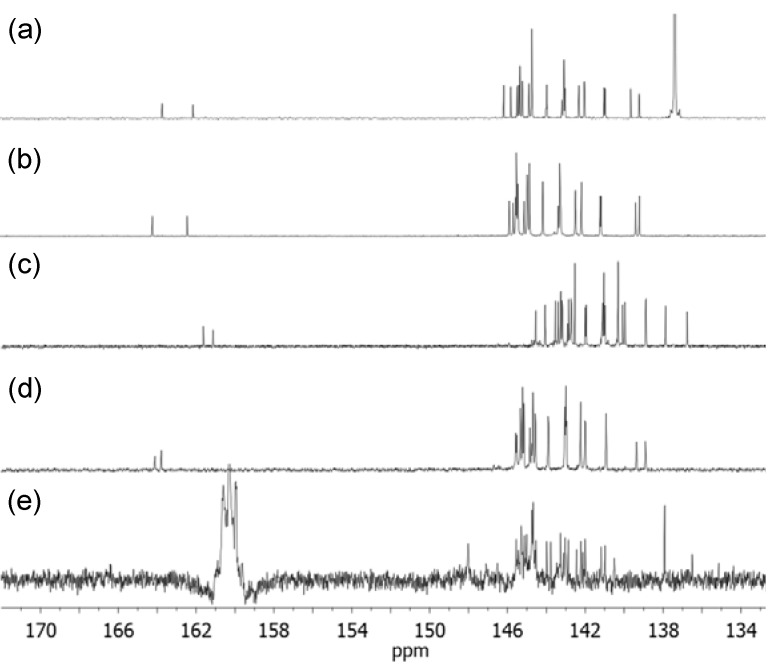
^13^C-NMR spectra of (**a**) **13** in toluene-*d*_8_; (**b**) **14** in CDCl_3_; (**c**) **15** in THF-*d*_8_–CS_2_; (**d**) **16** in CDCl_3_; and (**e**) **1′** in CDCl_3_.

In the case ^1^H-NMR spectroscopic analyses, we first well-characterized the arm structures ME_1_(*t*-C_4_) (**11**, Figure 2a) and ME_3_(*t*-C_4_) (**12**) by identification of all protons (H_α_, H_e_, H_f_, H_g_, and H_i_ indicated in Scheme 2) with their assignments to peaks shown in the spectra. Attachment of **11** on C_60_ leading to a monoadduct C_60_[>ME_1_(*t*-C_4_)] (**13**) was evident by the disappearance of a H_α_ proton peak with the chemical shift at *δ* 3.33. It was also accompanied with up-fielded shifts of two proton peaks to *δ* 3.06 and 3.26, corresponding to the chemical shift of terminal methoxy protons H_g _and ethylene oxide protons H_f_, in toluene-*d*_8_ from *δ* 3.34 and 3.56 of **11** in CDCl_3_, respectively, reflecting partly the solvent effect. Hydrolysis of **13** to C_60_(>ME_1_H) (**15**) was also apparent by the loss of *t*-butyl protons at *δ* 1.55, as shown in Figure 2b and Figure 2c, accompanied with down-fielded shifts of proton peaks (H_g_, H_f_, and H_e_) to *δ* 3.34, 3.71, and 4.59, respectively, in THF-*d*_8_ showing a larger solvent effect. Monoadduct structures of **13** and **14** were also confirmed by the detection of well-defined characteristic fullerenyl sp^2^ carbon peaks in their ^13^C-NMR spectra (Figure 3b) showing a group of 26 and 27 peaks, respectively, each accounted for two carbons and a group of 6 single carbon peaks for **13** (two of these single carbon peaks may be derived from the slightly unsymmetrical environment around the malonate moiety) and 4 single carbon peaks for **14** in the region of *δ *135–150, consistent with a *C*_v_ molecular symmetry for both **13** and **14**. It also displayed two clear carbonyl carbon peaks at *δ *163.45 (O=**C**-O-*tert*-butyl) and 161.86 (O=**C**-O–) in toluene-*d*_8_, for **13** and the corresponding peaks at *δ *163.95 and 162.16 in CDCl_3_ for **14**. Evidence of the acid formation in the structure of **15** and C_60_(>ME_3_H) (**16**) was given by the solubility change and up-fielded shifts of the carbonyl chemical-shift to *δ* 161.13 (O=**C**-OH) and 161.62 (O=**C**-O–) in THF-*d*_8_–CS_2_ (2:1) for **15 **(Figure 3c), partly due to the solvent effect. More clear changes of carbonyl carbon peak positions were observed for the case of **16** showing down-fielded shifts to *δ *163.79 (O=**C**-OH) and 164.12 (O=**C**-O–) (Figure 3d) from those of **14** in the same solvent, consistent with the corresponding structural modification. 

In addition, the spectra of both compounds **15** and **16** displayed a group of 27 peaks each accounted for two sp^2^ fullerenyl carbons and four single sp^2^ carbon peaks in the region of *δ *135–145 provided the further confirmation of a *C*_v_ molecular symmetry of C_60_(>ME_1_H) and C_60_(>ME_3_H), respectively, as monoadducts without change during the chemical reaction. 

Attachment of hexa(aminoethyl)amine C_3_N_6_^+^ arm to the malonic acid moiety of C_60_(>ME_1_H) leading to the structure of C_60_(>ME_1_N_6_^+^C_3_) **1′** and subsequently **1** was confirmed by the analyses of both infrared and ^1^H-NMR spectra. The latter of **1′** showed proton peaks corresponding to the C_3_N_6_^+^ arm moiety with overlap of a number of terminal ammonium-propyl *α*-protons (H_c_), *α*-protons to amide group (H_d_), glycol ether protons (H_f_), and terminal methoxy protons (H_g_) giving broad multiplet peaks in the region of *δ *3.0–4.25 in CDCl_3_–toluene-*d*_8_ (Figure 2d). The second broad multiplet peaks in the region of *δ *4.25–4.75 were assigned to ammonium-ethyl protons (H_c__′_) and *α*-protons to the ester group (H_e_). In the case of **1**, chemical shifts of similar groups of protons (H_c_, H_d_, H_f_, and H_g_) and (H_c__′_, H_e_, and ammonium-methyl protons H_h_) were assigned to those of broad multiplet peaks at *δ *3.0–3.75 and 3.75–4.5, respectively, in DMSO-*d*_6_–toluene-*d*_8_ (2:1), as shown in Figure 2(e). Interestingly, ^13^C-NMR spectrum of C_60_(>ME_1_N_6_^+^C_3_) **1′** (Figure 3e) showed a group of 27 peaks each accounted for two sp^2^ fullerenyl carbons and four single sp^2 ^carbon peaks (two of them having an identical chemical shift) in the region of *δ *135−150 that provided a clear confirmation of a *C*_v_ molecular symmetry of C_60_(>ME_1_N_6_^+^C_3_) monoadduct.

## 3. Experimental

### 3.1. Materials

The reagents 2-methoxyethanol, triethylene glycol monomethyl ether, 2,2-dimethyl-1,3-dioxane-4,6-dione (Meldrum’s acid), *N*-hydroxysuccinamide (NHS), *N*,*N*′-dicyclohexyl carbodiimide (DCC), iodomethane, carbon tetrabromide (CBr_4_), 1,8-diazabicyclo[5,4,0]-undec-7-ene (DBU), 2-methyl-2-propanol, trifluoroacetic acid (TFA), thionyl chloride (SOCl_2_), K_2_CO_3_, *γ*-butyrolactone (GBL), BF_3_·Et_2_O, triethylamine, and pyridine were purchased from Aldrich Chemicals and used without further purification. Malonyl chloride was purchased from TCI America. A C_60_ sample with a purity of 99.0% was used. Sodium sulfate was employed as a drying agent. Solvents were routinely distilled before use. 

### 3.2. Spectroscopic Measurements

Infrared spectra were recorded as KBr pellets on a Thermo Nicolet Avatar 370 FT-IR spectrometer. ^1^H-NMR and ^13^C-NMR spectra were recorded on a Bruker Avance Spectrospin-500 spectrometer. UV-Vis spectra were recorded on a Perkin Elmer Lambda 750 UV-Vis-NIR Spectrometer. MALDI-mass spectra were recorded on a WATERS Micromass MALDI-TOF mass spectrometer. Elemental analysis was taken by Galbraith Laboratories, Inc. (Knoxville, TN, USA).

### 3.3. Synthetic Procedures

#### 3.3.1. Synthesis of Malonic Acid Methoxyethyleneglycol Ester, ME_1_ (**4**)

A mixture of 2-methoxyethanol (1.0 g, 13.1 mmol) and 2,2-dimethyl-1,3-dioxane-4,6-dione (**3**, Meldrum’s acid, 1.9 g, 13.2 mmol) was stirred under an Ar atmosphere over a period of 12 h at 95 °C. The reaction mixture was cooled to room temperature, treated with aqueous sodium carbonate solution (5%), and washed with diethyl ether. The resulting aqueous layer was subsequently treated with dil. HCl (2.0 N) and extracted with ethyl acetate (50 mL). The ethyl acetate solution was dried over Na_2_SO_4_ and concentrated on rotary evaporator to give the product ME_1_ (**4**) in 95% yield (2.0 g). Spectroscopic data: FT-IR (KBr) *υ*_max_ 3060 (w), 2880 (w), 2814 (w), 1727 (vs), 1458 (w), 1399 (m), 1318 (s), 1249 (s), 1134 (s), 1100 (s), 1039 (m), 952 (w), 848 (s), and 755 (m) cm^−1^; ^1^H-NMR (500 MHz, CDCl_3_, ppm) *δ *10.60 (s, br, 1H), 4.31 (t, *J* = 4.63 Hz, 2H), 3.62 (t, *J* = 4.63 Hz, 2H), 3.45 (s, 2H), and 3.38 (s, 3H).

#### 3.3.2. Synthesis of Malonic Acid Methoxytriethyleneglycol Ester, ME_3_ (**5**)

A mixture of triethylene glycol monomethyl ether (1.0 g, 6.1 mmol) and 2,2-dimethyl-1,3-dioxane-4,6-dione (**3**, Meldrum’s acid, 0.9 g, 6.2 mmol) was stirred under Ar atmosphere over a period of 12 h at 90 °C. The reaction mixture was cooled to room temperature, treated with aqueous sodium carbonate solution (5%), and washed with diethyl ether. The resulting aqueous layer was subsequently treated with dil. HCl (2.0 N) and extracted with ethyl acetate (50 mL). The ethyl acetate solution was dried over Na_2_SO_4_ and concentrated on rotary evaporator to give the product ME_3_ (**5**) in 90% yield (1.37 g). Spectroscopic data: FT-IR (KBr) *υ*_max_ 3056 (w), 2880 (w), 2817 (w), 1728 (vs), 1455 (w), 1394 (m), 1318 (s), 1251 (s), 1134 (vs), 1098 (vs), 1034 (m), 952 (w), 847 (s), and 753 (m) cm^−1^; ^1^H-NMR (500 MHz, CDCl_3_, ppm) *δ *9.65 (s, br, 1H), 4.32 (t, *J* = 4.50 Hz, 2H), 3.70 (t, *J* = 4.50 Hz, 2H), 3.65–3.55 (m, 8H), 3.42 (s, 3H), and 3.41 (s, 2H).

#### 3.3.3. Synthesis of Methoxyethyleneglycol-[*N,N′,N,N,N,N*-hexapropyl-hexa(aminoethyl)amino]-malonamide Ester, ME_1_N_6_C_3_ (**7**)

A mixture of malonic acid methoxyethyleneglycol ester **4** (0.5 g, 3.08 mmol), *N*-hydroxysuccinamide (0.35 g, 3.08 mmol), and *N*,*N*′-dicyclohexyl carbodiimide (DCC, 0.63 g, 4.0 mmol) in anyhydrous tetrahydrofuran (20 mL) were stirred under N_2_ atmosphere over a period of 12 h at ambient temperature. The resulting white solids of *N*,*N*′-dicyclohexyl urea byproduct were filtered off and the filtrate was taken into a second round-bottom flask containing *N,N′,N,N,N,N-*hexapropyl-hexa(aminoethyl)amine **6** (1.49 g, 3.08 mmol). The mixture was stirred under N_2_ atmosphere for an additional period of 12 h. At the end of the reaction, the solvent was removed on rotavap. To this residue, ice-cold hexane–dichloromethane (1:1, 15 mL) was added followed by filtration to remove further white solids of *N*-hydroxysuccinamide. The filtrate was washed with aqueous sodium carbonate (5%) solution (10 mL). The organic layer was then dried and concentrated to give ME_1_N_6_C_3_ (**7**) as yellow liquid in 82% yield (1.59 g). Spectroscopic data: ESI–MS (rel. intensity) *m*/*z *568, 570 (M–CH_3_OCH_2_CH_2_, 75%), 571, 629 (M^+^), 630 (MH^+^, 100%), 631, 646, 666, 670, 688, 700, 715 [MH^+^+CH_2_CH_2_N(CH_2_CH_2_CH_3_), 48%], 716, 755, 757, 772, 773, 775, 800 (MH+2[CH_2_CH_2_N(CH_2_CH_2_CH_3_)], 15%), 801, 832, 860, 1150, 1210, 1212 (the dimer mass–CH_3_OCH_2_), 1236, and 1252 (from the dimer ion mass); FT-IR (KBr) *υ*_max_ 3259 (w), 2956 (vs), 2932 (s), 2872 (m), 2808 (m), 1742 (s), 1660 (s), 1550 (w), 1459 s), 1273 (m), 1128 (m), 1074 (s), 1031 (m), and 742 (w) cm^−1^; ^1^H-NMR (500 MHz, CDCl_3_, ppm) *δ *4.29 (t, *J* = 4.25 Hz, 2H), 3.61 (t, *J* = 4.25 Hz, 2H), 3.40 (s, 3H), 3.34–3.32 (m, 4H), 2.38–2.57 (m, 30H), 1.47–1.44 (m, 12H), and 0.90–0.87 (t, *J* = 7.15 Hz, 18H); ^13^C-NMR (500 MHz, CDCl_3_, ppm) *δ *168.78 (–**C**=O), 164.65 (O=**C**-NH–), 70.06, 64.15, 58.90, 57.35, 57.29, 57.13, 56.83 (4C), 56.72, 56.55, 53.65, 53.00, 52.81, 52.58, 52.46, 52.00, 41.60, 37.58, 20.41, 20.39, 20.37, 20.32 (2C), 20.18, 11.90 (3C), 11.87 (2C), and 11.74. 

#### 3.3.4. Synthesis of Methoxy-tri(ethyleneglycol)-[*N,N′,N,N,N,N*-hexapropyl-hexa(aminoethyl)-amino]-malonamide Ester, ME_3_N_6_C_3_ (**8**)

A mixture of malonic acid methoxytriethyleneglycol ester **5** (1.0 g, 4.0 mmol), *N*-hydroxysuccinamide (0.45 g, 4.0 mmol), and *N,N′*-dicyclohexyl carbodiimide (0.82 g, 4.0 mmol) in anyhydrous tetrahydrofuran (30 mL) were stirred under N_2_ atmosphere for a period of 12 h at ambient temperature. The resulting white solids of *N,N′*-dicyclohexyl urea byproduct were filtered off and the filtrate was taken into a second round-bottom flask containing *N,N′,N,N,N,N*-hexapropyl-hexa(aminoethyl)amine **6** (1.94 g, 3.9 mmol). The mixture was stirred under N_2_ atmosphere for an additional period of 12 h. At the end of the reaction, the solvent was removed on rotavap. To this residue, ice-cold hexane–dichloromethane (1:1, 20 mL) was added followed by filtration to remove white solids of *N*-hydroxysuccinamide. The filtrate was washed with aqueous sodium carbonate (5%) solution (10 mL). The organic phase was then dried and concentrated to give ME_3_N_6_C_3_ (**8**) as yellow liquid in 80% yield (2.29 g). Spectroscopic data: ESI–MS (rel. intensity) *m*/*z *512, 568 [M–CH_3_O(CH_2_CH_2_O)_2_CH_2_, 65%], 660 (M–CH_3_OCH_2_CH_2_, 55%), 716, 718 (MH^+^, 100%), 734, 776, 803 [MH^+^+CH_2_CH_2_N(CH_2_CH_2_CH_3_) from the dimer mass, 46%], 819, 860, 888 (MH^+^+2[CH_2_CH_2_N(CH_2_CH_2_CH_3_)] from the dimer mass, 8%), 944, 951, 1008, 1090, 1226 (the dimer mass–[MeO(EG)_3_-CO], 3%), 1238, 1282, 1340, 1430, and 1434 (the dimer ion); FT-IR (KBr) *υ*_max_ 3328 (w), 2952 (m), 2928 (m), 2862 (m), 2805 (m), 1736 (vs), 1667 (s), 1533 (m), 1456 (m), 1400 (m), 1379 (m), 1245 (m), 1104 (vs), 1032 (w), 938 (w), and 732 (m) cm^−1^; ^1^H-NMR (500 MHz, CDCl_3_, ppm) *δ *4.22 (t, *J *= 4.68 Hz,2H), 3.65 (t, *J* = 4.68 Hz, 2H), 3.58–3.60 (m, 6H), 3.48 (t, *J* = 4.60 Hz, 2H), 3.32 (s, 5H), 3.26 (s, br, 2H), 2.31−2.48 (m, 30H), 1.40–1.38 (m, 12H), and 0.82−0.79 (t, *J* = 6.95 Hz, 18H); ^13^C-NMR (500 MHz, CDCl_3_, ppm) *δ *168.83 (–**C**=O), 164.70 (O=**C**-NH–), 71.86 (2C), 70.53, 70.51, 68.74, 64.30, 58.95, 57.32 (2C), 57.14, 56.83 (4C), 56.72, 56.53, 53.64, 53.41, 52.98 (2C), 52.80, 52.45, 41.54, 37.55, 20.40, 20.37, 20.31 (3C), 20.24, 11.91 (3C), 11.88 (2C), and 11.76.

#### 3.3.5. Synthesis of Methoxyethyleneglycol-[*N,N′,N,N,N,N*-hexapropyl-hexa(aminoethyl)amino]-malonamide Ester Quaternary Methyl Ammonium Salt, ME_1_N_6_^+^C_3_ (**9**)

A solution of malonamide ester **7** (1.0 g, 1.6 mmol) in anhydrous chloroform–DMF (10:1) was added iodomethane (6.0 mL, excess, in portions) and stirred at 45 °C for a period of 3.0 days. At the end of quaternization, the solvent was evaporated to afford ME_1_N_6_^+^C_3_ (**9**) in 94% yield (2.0 g). Spectroscopic data: FT-IR (KBr) *υ*_max_ 3444 (s), 3236 (m), 2968 (vs), 2937 (vs), 2877 (m), 1735 (s), 1667 (s), 1536 (m), 1459 (vs), 1331 (w), 1126 (m), 1032 (m), 948 (m), 872 (w), 750 (vs), and 661 (m) cm^−1^; ^1^H-NMR (500 MHz, DMSO-*d*_6_, ppm) *δ *3.80–4.25 (m, br, 16H), 3.22–3.80 (m, br, 41H), 1.50–1.85 (m, br, 12H), and 0.88-0.99 (m, br, 18H); ^13^C-NMR (500 MHz, DMSO-*d*_6_, ppm) *δ *168.09 (-**C**=O), 166.69 (O=**C**-NH–), 79.90, 79.70, 70.01, 64.28 (4C), 63.59 (5C), 58.54, 55.54 (4C), 53.96 (2C), 49.37 (3C), 48.85 (2C), 42.62, 16.27 (2C), 15.95 (4C), 10.94 (2C), 10.82 (2C), and 10.72 (2C) (one peak is covered by DMSO peaks). Anal. Calcd for C_34_H_72_N_6_O_4_∙4.5CH_3_I∙2H_2_O (based on 90% quarternization on average): C, 35.47; H, 6.92; N, 6.45; I, 43.80; O, 7.36%. Found: C, 34.72; H, 6.82; N, 6.46; I, 42.31%.

#### 3.3.6. Synthesis of Methoxy-tri(ethyleneglycol)-[*N,N′,N,N,N,N*-hexapropyl-hexa(aminoethyl)-amino]-malonamide Ester Quaternary Methyl Ammonium Salt, ME_3_N_6_^+^C_3_ (**10**)

A solution of malonamide ester **8** (0.50 g, 0.70 mmol) in anhydrous chloroform–DMF (10:1) was added iodomethane (3.0 mL, excess, in portions) and stirred at 45 °C for a period of 3.0 days. At the end of quaternization, the solvent was evaporated to afford ME_3_N_6_^+^C_3_ (**10**) in 92% yield (0.92 g). Spectroscopic data: ESI–MS (rel. intensity) *m*/*z* 448 (100%), 503, 588 (40%), 731 (M^+^–5I–4CH_3_, 6%), 760, 815, 872, 873 (M^+^–4I–3CH_3_, 8%), 930, 1014, 1016 (M^+^–3I–2CH_3_, 13%), 1074, 1099, 1157 (M^+^–2I–CH_3_, 37%), 1198, 1216, 1299, 1300 (M^+^–I, 22%), 1385, 1391, 1392, 1442, 1443, and 1444 (M^+^+H_2_O); FT-IR (KBr) *υ*_max_ 3436 (vs), 3255 (m), 3006 (m), 2968 (s), 2935 (s), 2877 (m), 1736 (s), 1665 (s), 1634 (s), 1545 (w), 1459 (s), 1383 (w), 1351 (w), 1332 (w), 1271 (w), 1200 (m), 1110 (s), 1034 (m), 950 (m), 877 (w), 757 (w), and 600 (m) cm^−1^; ^1^H-NMR (500 MHz, DMSO-*d*_6_, ppm) *δ *3.80–4.25 (m, br, 16H), 2.90–3.80 (m, br, 49H), 1.50–1.80 (m, br, 12H), and 0.85–0.99 (m, br, 18H);^ 13^C-NMR (500 MHz, DMSO-*d*_6_, ppm) *δ *168.13 (–**C**=O), 166.69 (O=**C**-NH–), 71.72, 70.23, 70.18, 70.03, 68.57, 63.56 (9C), 54.79 (6C), 49.05 (5C), 42.62, 16.18 (2C), 15.97 (4C), 10.95 (2C), 10.82 (3C), and 10.70 (one peak is covered by DMSO peaks). Anal. Calcd for C_38_H_80_N_6_O_6_∙4CH_3_I∙3H_2_O (based on 80% quarternization on average): C, 37.67; H, 7.38; N, 6.28; I, 37.91; O, 10.76%. Found: C, 36.10; H, 7.03; N, 6.81; I, 37.90%.

#### 3.3.7. Synthesis of Pentacationic Methoxyethyleneglycol-(20-oxo-4,7,10,13,16-pentapropyl-4,7,10,13, 16,19-hexaaza-nonadecan-19-yl)[60]fullerenyl Malonate Quaternary Methyl Ammonium Salt, C_60_(˃ME_1_N_6_^+^C_3_) (**1**)

Finely divided [60]fullerene (0.94 g, 1.30 mmol, more than two-fold excess to allow the formation of monoadduct only) was taken into a round bottom flask and added anhydrous toluene (700 mL) under nitrogen. The solution was stirred for 12 h at ambient temperature to ensure complete dissolution of C_60_. To the resulting purple-colored solution added carbon tetrabromide (0.19 g, 0.57 mmol) followed by a solution of the compound **9** (0.70 g, 0.52 mmol) in anhydrous DMF (100 mL). The solution mixture was stirred for an additional 30 min and added slowly 1.8-diazabicyclo[5.4.0]-undec-7-ene (DBU, 0.17 g, 1.15 mmol) over a period of 45 min. The color of solution slowly turns into brown in a reaction period of 8.0 h. The solution was then concentrated on rotavap to roughly 100 mL. Upon the addition of methanol to this concentrated solution, the crude product was precipitated as brown solids which were collected via centrifugation. Unreacted C_60_ in the crude solids was removed by repeated washings with toluene (5 × 100 mL) until no color in the washing solution or filtrate. The remaining product of C_60_(>ME_1_N_6_^+^C_3_) (**1**) was obtained as brown solids in 55% yield (0.59 g, after recovered C_60_). Spectroscopic data: FT-IR (KBr) *υ*_max_ 3383 (vs), 2963 (m), 2932 (m), 2870 (m), 2814 (w), 1739 (s), 1686 (s), 1625 (s), 1455 (vs), 1426 (s), 1373 (w), 1240 (w), 1067 (s), 1031 (s), 947 (m), 728 (m), 575 (m), and 525 (vs, a characteristic band of C_60_monoadduct) cm^−1^; UV-Vis (DMF, cutoff at 268 nm, 2.0 × 10^–5^ M) *λ*_max_ 323 nm (shoulder peak); ^1^H-NMR [500 MHz, DMSO-d6–toluene-d8 (2:1), ppm] *δ *3.80–4.25 (m*,* br, 16H), 2.90–3.80 (m, br, 39H), 1.50–1.80 (m, br, 12H), and 0.88–0.99 (m, br, 18H). We found that electronic interferences of iodide anions in a high quantity with the fullerene cage or possible partial electron-transfer events prohibited the detection of fullerenyl carbon peaks (in low signal intensity).

#### 3.3.8. Synthesis of Pentacationic Methoxy-tri(ethyleneglycol)-(20-oxo-4,7,10,13,16-pentapropyl-4,7,10,13,16,19-hexaaza-nonadecan-19-yl)[60]fullerenyl Malonate Methyl Quaternary Ammonium Salt, C_60_(˃ME_1_N_6_^+^C_3_) (**2**)

Finely divided [60]fullerene (1.0 g, 1.40 mmol, more than two-fold excess to allow the formation of monoadduct only) was taken into a round bottom flask and added anhydrous toluene (700 mL) under nitrogen. The solution was stirred for 12 h at ambient temperature to ensure complete dissolution of C_60_. To the resulting purple-colored solution added carbon tetrabromide (0.17 g, 0.51 mmol) followed by a solution of compound **10** (0.65 g, 0.45 mmol) in anhydrous DMF (100 mL). The solution mixture was stirred for an additional 30 min of stirring and added slowly 1.8-diazabicyclo[5.4.0]-undec-7-ene (DBU, 0.15 g, 0.98 mmol) over a period of 45 min. The color of solution slowly turns into brown in a reaction period of 8 h. The solution was then concentrated on rotavap to roughly 100 mL. Upon the addition of methanol to this concentrated solution, the crude product was precipitated as brown solids which were collected via centrifugation. Unreacted C_60_ in the crude solids was removed by repeated washings with toluene (5 × 100 mL) until no color in the washing solution or filtrate. The remaining product of C_60_(>ME_3_N_6_^+^C_3_) (**2**) was obtained as brown solids in 50% yield (0.343 g, after recovered C_60_). Spectroscopic data: FT-IR (KBr) *υ*_max_ 3433 (vs), 3262 (s), 2963 (m), 2925 (m), 2868 (m), 2809 (w), 1736 (s), 1695 (m), 1630 (s), 1459 (s), 1384 (w), 1197 (m), 1107 (s), 1071 (s), 938 (m), 757 (w), and 525 (s, a characteristic band of C_60_monoadduct) cm^−1^; UV-Vis (DMF, cutoff at 268 nm, 2.0 × 10^−5^ M) *λ*_max_ 323 nm (shoulder peak); ^1^H-NMR [500 MHz, DMSO-d6–toluene-d8 (2:1), ppm] *δ *3.80–4.20 (m*,* br, 16H), 2.90–3.80 (m, br, 47H), 1.50–1.75 (m, br, 12H), and 0.88–0.99 (m, br, 18H). We found that electronic interferences of iodide anions in a high quantity with the fullerene cage or possible partial electron-transfer events prohibited the detection of fullerenyl carbon peaks (in low signal intensity). 

#### 3.3.9. Synthesis of *tert*-Butyl(2-methoxyethyl)malonate, ME_1_(*t*-C_4_) (**11**)

A mixture of malonic acid methoxyethyleneglycol ester **4** (1.0 g, 6.16 mmol), 2-methyl-2-propanol (0.54 g, 7.39 mmol), and *N*,*N*′-dicyclohexyl carbodiimide (DCC, 1.27 g, 6.16 mmol) in anyhydrous dichloromethane (20 mL) were stirred under atmospheric pressure of N_2_ over a period of 8.0 h at ambient temperature. The resulting white solid of *N*,*N*′-dicyclohexyl urea was filtered and the filtrate was washed with aqueous sodium carbonate solution (5%, 10 mL). The organic layer was then dried over sodium sulfate and concentrated on rotavap to give ME_1_(*t*-C_4_) (**11**) in 74% yield (1.0 g) as light yellow liquid. Spectroscopic data: FT-IR (KBr) *υ*_max_ 2980 (w), 2930 (w), 2880 (w), 2824 (w), 1748 (s), 1726 (vs), 1455 (w), 1406 (w), 1393 (w), 1368 (m), 1330 (m), 1281 (m), 1250 (m), 1199 (m), 1127 (vs), 1100 (m), 1037 (s), 966 (m), 864 (m), 838 (m), 759 (w), and 738 (w) cm^−1^; ^1^H-NMR (500 MHz, CDCl_3_, ppm) *δ *4.27 (t, *J* = 3.94 Hz, 2H), 3.56 (t, *J* = 3.94 Hz, 2H), 3.34 (s, 3H), 3.29 (s, 2H, H_α_), and 1.43 (s, 9H).

#### 3.3.10. Synthesis of *tert*-Butyl(methoxy-triethyleneglycol)malonate, ME_3_(*t*-C_4_) (**12**)

A mixture of malonic acid methoxytriethyleneglycol ester **5** (3 g, 11.98 mmol), 2-methyl-2-propanol (1.06 g, 14.38 mmol), and *N*,*N*′-dicyclohexyl carbodiimide (2.47 g, 11.98 mmol) in anyhydrous dichloromethane (20 mL) were stirred under atmospheric pressure of N_2_ over a period of 8.0 h at ambient temperature. The resulting white solid of *N*,*N*′-dicyclohexyl urea was filtered and the filtrate was washed with aqueous sodium carbonate solution (5%, 10 mL). The organic layer was then dried over sodium sulfate and concentrated on rotavap to give ME_3_(*t*-C_4_) (**12**) in 77% yield (2.85 g) as light yellow liquid. Spectroscopic data: FT-IR (KBr) *υ*_max_ 2973 (w), 2930 (w), 2876 (w), 2817 (w), 1748 (s), 1727 (vs), 1455 (w), 1393 (w), 1368 (m), 1330 (m), 1282 (m), 1250 (m), 1198 (m), 1134 (vs), 1104 (vs), 1040 (m), 966 (m), 864 (m), 759 (w), and 735 (w) cm^−1^; ^1^H-NMR (500 MHz, CDCl_3_, ppm) *δ *4.26 (t, *J* = 4.50 Hz, 2H), 3.68 (t, *J* = 4.50 Hz, 2H), 3.63 (m, 6H), 3.52 (t, *J* = 4.50 Hz, 2H), 3.35 (s, 3H), 3.29 (s, 2H, *α*-H), and 1.44 (s, 9H).

#### 3.3.11. Synthesis of *tert*-Butyl(2-methoxyethyl)[60]fullerenyl Malonate, C_60_[˃ME_1_(*t*-C_4_)] (**13**)

Finely divided [60]fullerene (1.23g, 1.70 mmol) was taken into a round bottom flask and added anhydrous toluene (850 mL) and 1,2-dichlorobenzene (30 mL) under nitrogen. The solution was stirred for 1.0 h at ambient temperature to ensure complete dissolution of C_60_. To the resulting purple-colored solution was added carbon tetrabromide (0.50 g, 1.51 mmol) followed by a solution of *tert*-butyl(2-methoxyethyl)malonate (**11**, 0.30 g, 1.37 mmol) in anhydrous toluene (10 mL). The solution mixture was stirred for an additional 30 min and added slowly 1.8-diazabicyclo[5.4.0]-undec-7-ene (DBU, 0.44 g, 2.88 mmol) over a period of 1.0 h. The color of solution slowly turned into brown in a reaction period of 12 h. The solution was then concentrated on a rotavap. The resulting crude product was purified using column chromatography with silica gel as the stationary phase and toluene–ethyl acetate (20:1) as eluent, giving the isolation of *tert*-butyl(2-methoxyethyl)[60]fullerenyl malonate (**13**), C_60_[>ME_1_(*t*-C_4_)], as brown solids in 78% yield (1.00 g). Spectroscopic data: FT-IR (KBr) *υ*_max_ 3442 (br, s), 2972 (w), 2917 (w), 2873 (w), 2814 (w), 1739 (vs), 1645 (m), 1426 (m), 1390 (w), 1366 (m), 1268 (s), 1232 (vs), 1179 (m), 1151 (s), 1110 (m), 1059 (m) 1026 (m), 828 (w), 738 (w), 704 (w), 577 (m), 551 (m), and 525 (vs, a characteristic band of C_60_ monoadduct) cm^−1^; ^1^H-NMR (500 MHz, toluene-*d*_8_, ppm) *δ *4.28 (t, *J* = 4.55 Hz, 2H), 3.26 (t, *J* = 4.55 Hz, 2H), 3.06 (s, 3H), and 1.55 (s, 9H); ^13^C-NMR (500 MHz, toluene-*d*_8_, ppm) *δ *163.45 (O=**C**-O-*tert*-butyl), 161.86 (O=**C**-O–), 145.89 (2C), 145.53 (2C), 145.22 (2C), 145.13 (2C), 145.06 (3C), 144.95 (2C), 144.93 (2C), 144.60 (2C), 144.59 (2C), 144.46 (3C), 144.45 (4C), 143.71 (2C), 143.68 (2C), 142.91 (C), 142.89 (C), 142.83 (2C), 142.81 (3C), 142.80 (3C), 142.74 (2C), 142.03 (2C), 142.02 (2C), 141.76 (2C), 141.74 (2C), 140.74 (2C), 140.68 (2C), 139.37 (2C), 138.93 (2C), 84.13, 72.07 (fullerenyl sp^3^ carbons, 2C), 69.74, 65.49, 58.00, 53.45, and 27.49 (3C).

#### 3.3.12. Synthesis of *tert*-Butyl(methoxy-triethyleneglycol)[60]fullerenyl Malonate, C_60_[˃ME_3_(*t*-C_4_)] (**14**)

Finely divided [60]fullerene (1.86 g, 2.59 mmol) was taken into a round bottom flask and added anhydrous toluene (850 mL) and 1,2-dichlorobenzene (50 mL) under nitrogen. The solution was stirred for 1.0 h at ambient temperature to ensure complete dissolution of C_60_. To the resulting purple-colored solution was added carbon tetrabromide (0.75 g, 2.27 mmol) followed by a solution of *tert*-butyl(methoxy-triethyleneglycol)malonate **12** (0.63 g, 2.07 mmol) in anhydrous toluene (10 mL). The solution mixture was stirred for an additional 30 min and added slowly 1.8-diazabicyclo[5.4.0]-undec-7-ene (DBU, 0.66 g, 4.35 mmol) over a period of 1.0 h. The color of solution slowly turned into brown in a reaction period of 12 h. The solution was then concentrated on rotavap and the resulting crude product was purified by column chromatography with silica gel as the stationary phase and toluene−ethyl acetate (9:1) as eluent to afford C_60_[>ME_3_(*t*-C_4_)] (**14**) as brown solids in 75% yield (1.60 g). Spectroscopic data: FT-IR (KBr) *υ*_max_ 3421 (br, s), 2967 (w), 2914 (w), 2864 (w), 2814 (w), 1740 (s), 1634 (m), 1456 (m), 1426 (m), 1392 (m), 1368 (m), 1269 (s), 1253 (s), 1226 (s), 1180 (m), 1154 (s), 1108 (m), 1029 (m), 845 (m), 737 (m), 702 (m), 669 (m), 576 (m), 550 (m), and 525 (vs) cm^−1^; ^1^H-NMR (500 MHz, CDCl_3_, ppm) *δ *4.65 (t, *J* = 4.60 Hz, 2H), 3.90 (t, *J* = 4.60 Hz, 2H), 3.73 (t, *J* = 4.60 Hz, 2H), 3.65–3.72 (m, 4H), 3.56 (t, *J* = 4.60 Hz, 2H), 3.39 (s, 3H), and 1.70 (s, 9H); ^13^C-NMR (500 MHz, CDCl_3_, ppm) *δ *163.95 (O=**C**-O-*tert*-butyl), 162.16 (O=**C**-O–), 145.61 (2C), 145.41 (2C), 145.29 (2C), 145.25 (4C), 145.22 (2C), 145.16 (3C), 144.85 (2C), 144.71 (C), 144.69 (3C), 144.66 (2C), 144.57 (4C), 143.89 (3C), 143.09 (2C), 143.08 (2C), 143.01 (4C), 142.99 (2C), 142.97 (2C), 142.23 (2C), 142.21 (2C), 141.91 (2C), 141.90 (2C), 140.94 (2C), 140.89 (2C), 139.11 (2C), 138.92 (2C), 85.16, 71.95 (fullerenyl sp^3^ carbons, 2C), 71.84, 70.68, 70.67, 70.65, 68.84, 66.07, 59.10, 53.06, and 28.07 (3C).

#### 3.3.13. Synthesis of 2-[60]Fullerenyl-3-(2-methoxyethoxy)-3-oxopropanoic Acid, C_60_(˃ME_1_H) (**15**)

The compound of [60]fullerenyl malonate **13** (0.5 g, 0.64 mmol) was taken into a round bottom flask containing anhydrous dichloromethane (50 mL) and purged with N_2_ for 15 minutes at ambient temperature. To this reaction mixture was added trifluoroacetic acid (30 mL, excess) and stirred for overnight at room temperature. At the end of the reaction, dichloromethane was removed on rotavap. Additional dichloromethane (3 × 15 mL) was added and removed on rotavap in order to fully eliminate an excessive amount of trifluoroacetic acid. The resulting residue was then washed with diethyl ether (2 × 15 mL) to afford C_60_(>ME_1_H) (**15**) as brown solids in 89% yield (0.50 g). Spectroscopic data: FT-IR (KBr) *υ*_max_ 3670 (br, s), 2977 (w), 2917 (w), 2878 (w), 2814 (w), 1792 (w), 1750 (s), 1708 (s), 1681 (m), 1541 (w), 1427 (m), 1390 (w), 1366 (m), 1267 (s), 1250 (s), 1231 (s), 1186 (m), 1114 (w), 1059 (w) 1020 (w), 864 (w), 743 (w), 701 (m), 572 (m), 552 (m), and 525 (vs, a characteristic band of C_60_ monoadduct) cm^−1^; ^1^H-NMR (500 MHz, THF-*d*_8_, ppm) *δ *4.59 (t, *J *= 4.50 Hz,2H), 3.71 (t, *J* = 4.50 Hz, 2H), and 3.34 (s, 3H); ^13^C-NMR [500 MHz, THF-d8–CS2 (2:1), ppm] *δ* 161.62 (O=**C**-O–), 161.13 (O=**C**-OH), 144.54 (2C), 144.06 (2C), 143.53 (2C), 143.39 (2C), 143.26 (2C), 143.25 (2C), 143.19 (2C), 143.16 (2C), 142.91 (C), 142.85 (2C), 142.78 (C), 142.73 (2C), 142.70 (2C), 142.53 (4C), 141.99 (2C), 141.95 (2C), 141.14 (C), 141.12 (C), 141.10 (2C), 141.04 (4C), 140.98 (2C), 140.31 (4C), 140.07 (2C), 139.95 (2C), 138.89 (2C) , 138.87 (2C), 137.87 (2C), 136.76 (2C), 70.48 (fullerenyl sp^3^ carbons, 2C), 68.14, 63.98, 56.30, and 51.46

#### 3.3.14 Synthesis of 2-[60]Fullerenyl-3-(methoxy-triethyleneglycol)-3-oxopropanoic Acid, C_60_(˃ME_3_H) (**16**)

The compound of [60]fullerenyl malonate **14** (0.75 g, 0.73 mmol) was taken into a round bottom flask containing anhydrous dichloromethane (50 mL) and purged with N_2_ for a period of 15 minutes at ambient temperature. To this reaction mixture was added trifluoroacetic acid (50 mL) and stirred for overnight at room temperature. At the end of the reaction, dichloromethane was removed on rotavap. Additional dichloromethane (3 × 20 mL) was added and removed on rotavap in ordered to fully eliminate an excessive amount of trifluoroacetic acid. The resulting residue was then washed with diethyl ether (3 × 20 mL) to afford C_60_(>ME_3_H) (**16**) as brown solids in 87% yield (0.62 g). Spectroscopic data: FT-IR (KBr) *υ*_max_ 3673 (br, s), 2917 (w), 2894 (w), 2870 (w), 2814 (w), 1788 (w), 1741 (vs), 1578 (w), 1532 (w), 1426 (m), 1383 (w), 1266 (m), 1230 (s), 1203 (m), 1180 (w), 1095 (m), 1060 (w) 845 (w), 698 (m), 579 (m), 549 (m), and 525 (vs) cm^−1^; ^1^H-NMR (500 MHz, CDCl_3_, ppm) *δ *4.70 (t*,J* = 4.55 Hz, 2H), 3.95 (t, *J* = 4.55 Hz, 2H), 3.80 (m, 6H), 3.76 (t, *J* = 4.60 Hz, 2H), and 3.59 (s, 3H); ^13^C-NMR (500 MHz, CDCl_3_, ppm) *δ *164.12 (O=**C**-O–), 163.79 (O=C-OH), 145.57 (2C), 145.52 (2C), 145.33 (3C), 145.32 (3C), 145.22 (4C), 145.15 (3C), 144.84 (2C), 144.75 (C), 144.68 (4C), 144.57 (2C), 144.54 (2C), 143.90 (2C), 143.88 (2C), 143.04 (3C), 142.99 (4C), 142.96 (2C), 142.23 (3C), 142.01 (2C), 141.97 (2C), 140.93 (3C), 140.92 (3C), 139.36 (2C), 138.90 (2C), 72.19, 71.88 (fullerenyl sp3 carbon, 2C), 70.89, 70.64, 70.33, 69.70, 68.34, 65.99, 58.78, and 52.51.

#### 3.3.15. Synthesis of Methoxyethyleneglycol-(20-oxo-4,7,10,13,16-pentapropyl-4,7,10,13,16,19-hexaaza-nonadecan-19-yl)[60]fullerenyl Malonate, C_60_(˃ME_1_N_6_C_3_) (**17**) and C_60_(&gt;ME_1_N_6_^+^C_3_) (**1′**)

The compound of 2-[60]fullerenyl-3-(2-methoxyethoxy)-3-oxopropanoic acid **15** (0.15 g, 0.17 mmol) was taken into a round bottom flask containing anhydrous tetrahydrofuran (20 mL). To this reaction mixture was added thionyl chloride (0.03 g, 2.55 mmol) under N_2 _atmosphere and refluxed for a period of 2.0 h. An excessive amount of thionyl chloride was removed on rotavap. Fresh anhydrous tetrahydrofuran (20 mL) was added. To this reaction solution was added slowly *N,N′,N,N,N,N-*hexapropyl-hexa(aminoethyl)amine **6** (0.08 g, 0.17 mmol) at 0 °C. It was warmed gradually to room temperature and stirred at this temperature for a period of 3.0 h. The resulting solution was concentrated on rotavap with the residue washed sequentially with hexane (10 mL), methanol (2 × 10 mL), and toluene (3 × 10 mL) to fully remove unreacted *N,N′,N,N,N,N-*hexapropyl-hexa(aminoethyl)amine and decarboxylated C_60_ byproducts. A relatively pure C_60_(>ME_1_N_6_C_3_) (**17**) was obtained in 47% yield (0.11 g). It was subsequently treated with CF_3_COOH to result in pentacationic quaternary ammonium−trifluoroacetate salt, C_60_(>ME_1_N_6_^+^C_3_) (**1′**), specifically, for NMR measurements. Spectroscopic data of the compound **17**: FT-IR (KBr) *υ*_max_ 3670 (br, s), 2977 (w), 2917 (w), 2878 (w), 2814 (w), 1792 (w), 1750 (s), 1708 (s), 1681 (m), 1541 (w), 1427 (m), 1390 (w), 1366 (m), 1267 (s), 1250 (s), 1231 (s), 1186 (m), 1114 (w), 1059 (w), 1020 (w), 864 (w), 743 (w), 701 (m), 572 (m), 552 (m), and 525 (vs, a characteristic band of C_60_monoadduct) cm^−1^. Spectroscopic data of the compound **1′**:^1^H-NMR [500 MHz, CDCl3–toluene-d8–TFA (3:1:1), ppm] *δ *4.22–4.68 (m, br, 2H), 3.90–4.05 (m, br, 2H), 3.22–3.75 (m, br, 35H), 1.28–1.36 (m, br, 12H), and 0.97 (m, br, 18H); ^13^C-NMR [500 MHz, CDCl_3_–toluene-*d*_8_–TFA (3:1:1), ppm] *d* 167.72 (O=**C**-O–), 166.46 (O=**C**-NH–), 148.00 (2C), 145.54 (2C), 145.30 (2C), 145.29 (2C), 145.24 (2C), 145.14 (2C), 145.02 (2C), 144.73 (4C), 144.68 (4C), 144.53 (2C), 143.98 (2C), 143.77 (2C), 143.27 (2C), 143.11 (C), 143.04 (2C), 143.01 (2C), 142.98 (C), 142.87 (2C), 142.44 (2C), 142.22 (2C), 142.12 (2C), 142.01 (2C), 141.17 (2C), 140.98 (2C), 140.51 (2C), 137.92 (4C), 136.50 (2C), 70.47, 70.23 (fullerenyl sp^3^ carbon, 2C), 70.08, 69.73, 64.65, 64.40, 58.94, 58.68, 38.43, 31.84, 22.04, 21.40, 16.66, and 10.10 (quaternary aminocarbon peaks were low in intensity). 

#### 3.3.16. Synthesis of Pentacationic Methoxyethyleneglycol-(20-oxo-4,7,10,13,16-pentapropyl-4,7,10,13,16,19-hexaaza-nonadecan-19-yl)[60]fullerenyl Malonate Quaternary Methyl Ammonium Salt, C_60_(˃ME_1_N_6_^+^C_3_) (**1**)

A solution of [60]fullerenyl malonate quaternary ammonium−trifluoroacetate salt **1′** (100 mg) in chloroform (50 mL) was neutralized with aqueous potassium carbonate (10%, 50 mL). The resulting [60]fullerenyl malonate **17** was then dissolved in a mixture of anhydrous chloroform (30 mL) and dimethylformamide (15 mL). To this reaction, an excess amount of iodomethane was added in several portions over the reaction period and stirred at 45 °C for 3.0 days. At the end of the reaction, the solvent was removed on rotavap to yield pentacationic C_60_(>ME_1_N_6_^+^C_3_) (**1**). Spectroscopic data: MALDI–TOF–MS (sinapic acid as the matrix, rel. intensity) *m*/*z* 673 (20%), 697 (50%), 721 (C_60_H^+^, 100%), 734 (80%), 746 (15%), 761 (10%), 772 (10%), 874 [C_60_(>H(C=O)NHCH_2_CH_2_N^+^-propylMe_2_), 20%], 1442 [(C_60_H)_2_^+^ cluster], 1565, 1634, 1709, 1769, 1851, 1930 (M^+^–I^−^); FT-IR (KBr) *υ*_max_ 3688 (br, s), 2918 (s), 2870 (m), 2840 (m), 2807 (w), 1784 (w), 1736 (vs), 1663 (s), 1574 (w), 1433 (m), 1383 (w), 1252 (w), 1187 (m), 1163 (s), 1126 (m), 1090 (m), 1031 (s), 842 (w), 762 (w), 704 (w), 661 (w), 569 (w), and 524 (vs, a characteristic band of C_60_ monoadduct) cm^−1^; UV-Vis (DMF, cutoff at 268 nm, 2.0 × 10^–5^ M) *λ*_max_ 323 nm (shoulder peak); ^1^H-NMR [500 MHz, DMSO-d6–toluene-d8 (2:1), ppm] *δ *3.80–4.25 (m, br, 16H), 2.90–3.80 (m, br, 39H), 1.50–1.80 (m, br, 12H), and 0.88–0.99 (m, br, 18H). We found that electronic interferences of iodide anions in a high quantity with the fullerene cage or possible partial electron-transfer events prohibited the detection of fullerenyl carbon peaks (in low signal intensity).

#### 3.3.17. Synthesis of Pentacationic Methoxy-tri(ethyleneglycol)-(20-oxo-4,7,10,13,16-pentapropyl-4,7,10,13,16,19-hexaaza-nonadecan-19-yl)[60]fullerenyl Malonate Methyl Quaternary Ammonium Salt, C_60_(˃ME_3_N_6_^+^C_3_) (**2**)

Synthesis of the compound **2** was carried out by using a similar procedure as that of **1** except methoxytriethyleneglycol ester was applied instead. Spectroscopic data: MALDI–TOF–MS (sinapic acid as the matrix, rel. intensity)*m*/*z* 698 (10%), 721 (C_60_H^+^, 100%), 735 (C_60_>H_2_, 20%), 749, 773, 782, 789, 809, 874 [C_60_(>H(C=O)NHCH_2_CH_2_N^+^-propylMe_2_)], 914, 940, 995, 1027, 1054, 1278 (w), 1395 (w), 1417 (vw), 1499 (vw), 1792 (vw), and 2019 (vw, MH^+^–I^−^); FT-IR (KBr) *υ*_max_ 3424 (vs), 2967 (m), 2925 (m), 2874 (m), 2824 (w), 1738 (s), 1681 (s), 1628 (s), 1454 (vs), 1429 (s), 1383 (m), 1064 (s), 1028 (s), 941 (m), 727 (m), 572 (m), and 524 (vs, a characteristic band of C_60_monoadduct) cm^−1^; UV-vis (DMF, cutoff at 268 nm, 2.0 × 10^−5^ M) *λ*_max_ 323 nm (shoulder peak); ^1^H-NMR [500 MHz, DMSO-d6–toluene-d8 (2:1), ppm] *δ *3.80–4.20 (m*,*br, 16H), 2.90–3.80 (m, br, 47H), 1.50–1.75 (m, br, 12H), and 0.88–0.99 (m, br, 18H). We found that electronic interferences of iodide anions in a high quantity with the fullerene cage or possible partial electron-transfer events prohibited the detection of fullerenyl carbon peaks (in low signal intensity). 

## 4. Conclusions

We have designed and synthesized two analogous pentacationic [60]fullerenyl monoadducts, C_60_(>ME_1_N_6_^+^C_3_) (**1**) and C_60_(>ME_3_N_6_^+^C_3_) (**2**), with variation of the methoxyethyleneglycol length. Each of these derivatives bears a well-defined number of cationic charges aimed to enhance and control their ability to target pathogenic Gram-positive and Gram-negative bacterial cells. The intrinsic nature of the high charge number and increased water-solubility of the precursor arm intermediates hindered the efficiency of their reactions with a highly hydrophobic C_60_ cage having low compatibility in polar solvents. Furthermore, consecutive ethylamino group linkage in a structure of *N,N′,N,N,N,N*-hexapropyl-hexa(aminoethyl)amine (C_3_N_6_) largely increased its electron-donating capability that gave complication in forming an insoluble partial charge-transfer complex with C_60_ during the reaction and workup procedures. After many attempts, we found a circumventive solution by the use of partially quaternized C_3_N_6_ prior to the reaction with C_60_ coupled with the modification of C_3_N_6 _arm using propyl groups, instead of methyl or ethyl groups, to provide a well-balanced hydrophobicity– hydrophilicity character of pentacationic precursor intermediates and better compatibility with the C_60_ cage moiety.

## Supplementary Materials

Supplementary materials can be accessed at: http://www.mdpi.com/1420-3049/17/5/5225/s1.
