# Supplementary material for: Synthesis and Characterization of Positively Charged Pentacationic [60]Fullerene Monoadducts for Antimicrobial Photodynamic Inactivation
Source: Molecules. 2012 May 7;17(5):5225–43. doi: 10.3390/molecules17055225 (PMC3361754; doi:10.3390/molecules17055225)

Supporting Information

Figure S1. ^{1}H-NMR spectra of (**a**) **7** in $CDCl_3$; (**b**) **9** in DMSO-d_6; (**c**) **1** in DMSO-d_6-toluene-d_8; (**d**) **8** in $CDCl_3$; (**e**) **10** in DMSO-d_6; and (**f**) **2** in DMSO-d_6-toluene-d_8.

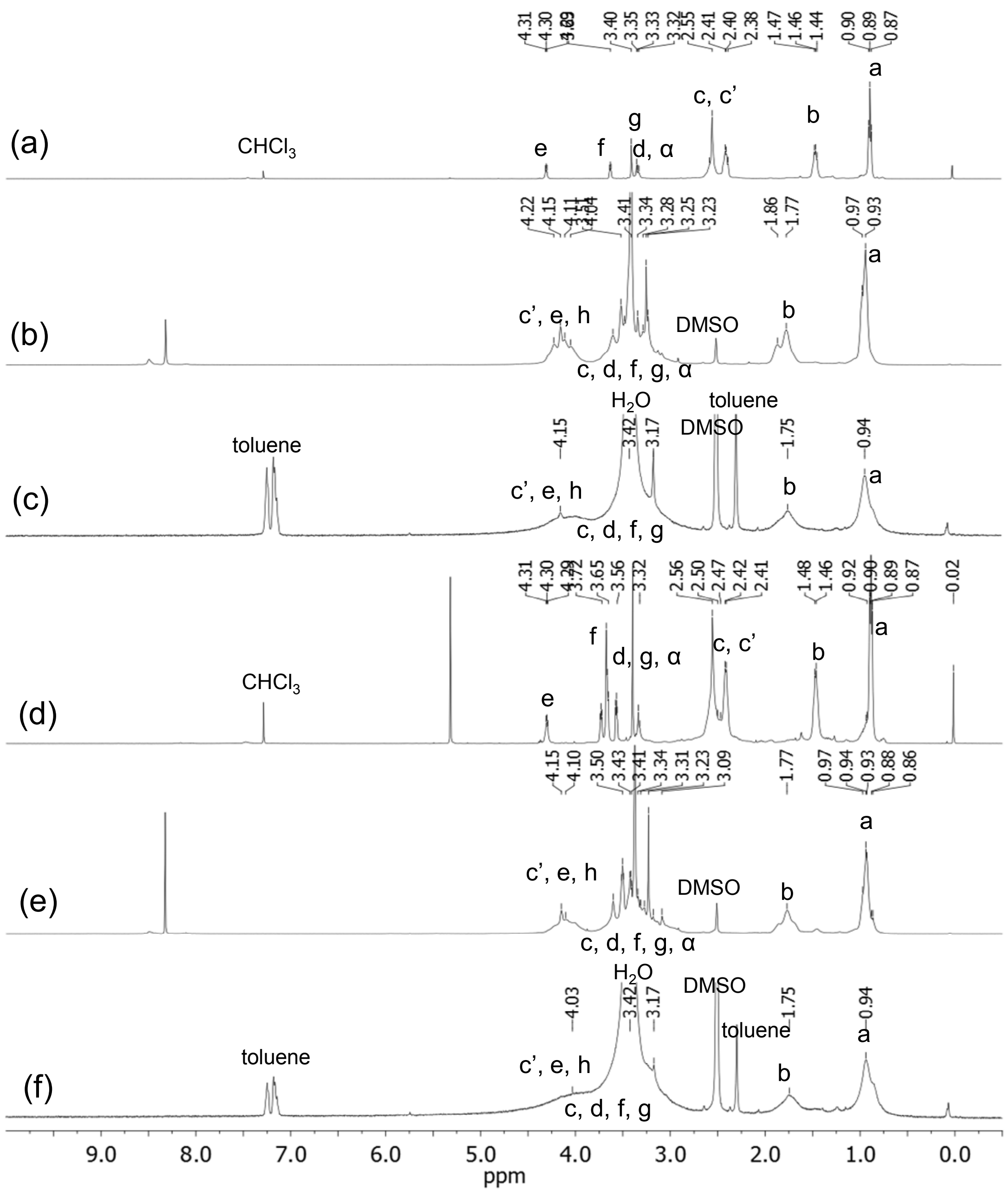

Al proton peaks of compounds **7**, **9**, **1** (Figure S1) were assigned with the corresponding proton numbers indicated on the compound structures shown in Scheme 1. Clear chemical shift assignment of all protons in the structure methoxyethyleneglycol-[*N,N',N,N,N,N*-hexapropyl-hexa(aminoethyl)amino]-malonamide ester, $ME_1N_6C_3$ (**7**) was used to correlate the chemical shift changes of $ME_1N_6^+C_3$ (**9**) and C_{60}>($ME_1N_6^+C_3$) (**1**) upon CH_3−I quaternization and C_{60} reaction. Accordingly, we assigned the peak at δ 0.89 (H_a), 1.44 (H_b), 2.38−2.53 (H_c and $H_{c'}$) of **7** to the chemical shift of methyl protons C**H**$_3$-CH$_2$-CH$_2$-N−, methylene protons CH$_3$-C**H**$_2$-CH$_2$-N−, and *N*-attached ethyleneamino –C**H**$_2$– protons; two peaks at δ 3.32 (H_d) and 4.29 (H_e) to the chemical shift of methylene protons of –C**H**$_2$-NH-C=O and

–C**H**$_2$-O-C=O, respectively; and two peaks at δ 3.61 (H$_f$) and 3.39 (H$_g$) to the methylene and methyl protons of −C**H**$_2$-O− and C**H**$_3$-O−, respectively. The chemical shift of malonate α-proton was found to be δ 3.33 as a singlet. Quaternization reaction of $ME_1N_6C_3$ (**7**) with iodomethane to $ME_1N_6^+C_3$ (**9**) resulted in the introduction of methylated quaternary amine arm in the structure that caused a large down-fielded shift of chemical shifts (more than 1.0 ppm) of all *N*-attached ethyleneamino –C**H**$_2$– protons at δ 2.38−2.53 to δ 3.22−4.25 (H$_c$, H$_{c'}$, and H$_h$) for quaternary ammonium methylene (–N$^+$-C**H**$_2$–), and additional methyl protons (–N$^+$-C**H**$_3$), with the overlap of H$_d$, H$_e$, H$_f$, H$_g$, and H$_\alpha$, as a broad band. Chemical shifts of all methyl or methylene proton peaks of C**H**$_3$-CH$_2$-CH$_2$-N$^+$− and CH$_3$-C**H**$_2$-CH$_2$-N$^+$− were also down-field shifted to δ 0.95 (H$_a$) and δ 1.50–1.85 (H$_b$), respectively.

Similar chemical shifts were made for assignments of all proton peaks of $ME_3N_6C_3$ (**8**) to those of $ME_1N_6C_3$ (**7**), as shown in Figure S1, except more complicated peaks at δ 3.55−3.71 for the methylene protons of −C**H**$_2$-O− (H$_f$). Other peaks are almost identical, those of all methyl or methylene proton peaks in the moieties of C**H**$_3$-O−, C**H**$_3$-CH$_2$-CH$_2$-N−, CH$_3$-C**H**$_2$-CH$_2$-N− and −C**H**$_2$-N− to δ 3.38 (H$_g$), 0.90 (H$_a$), δ 1.47 (H$_b$), and 2.40−2.54 (H$_c$ and H$_{c'}$) respectively. After methylated quaternization and the reaction with C_{60}, ^{1}H NMR spectrum of $C_{60}(>ME_3N_6^+C_3)$ (**2**) showed nearly identical chemical shifts as those of $C_{60}(>ME_1N_6^+C_3)$ (**1**), except a slight shift of chemical shifts of all methyl or methylene proton peaks in the moieties of C**H**$_3$-CH$_2$-CH$_2$-N−, CH$_3$-C**H**$_2$-CH$_2$-N−, −C**H**$_2$-N− (C**H**$_3$-N$^+$−) to δ 0.94 (H$_a$), δ 1.75 (H$_b$), and δ 2.90−4.20 (H$_c$, H$_{C'}$, H$_h$), respectively.

Figure S2. MALDI-TOF mass spectra of $C_{60}(>ME_1N_6^+C_3)$ (**1**) using sinapic acid as the matrix.

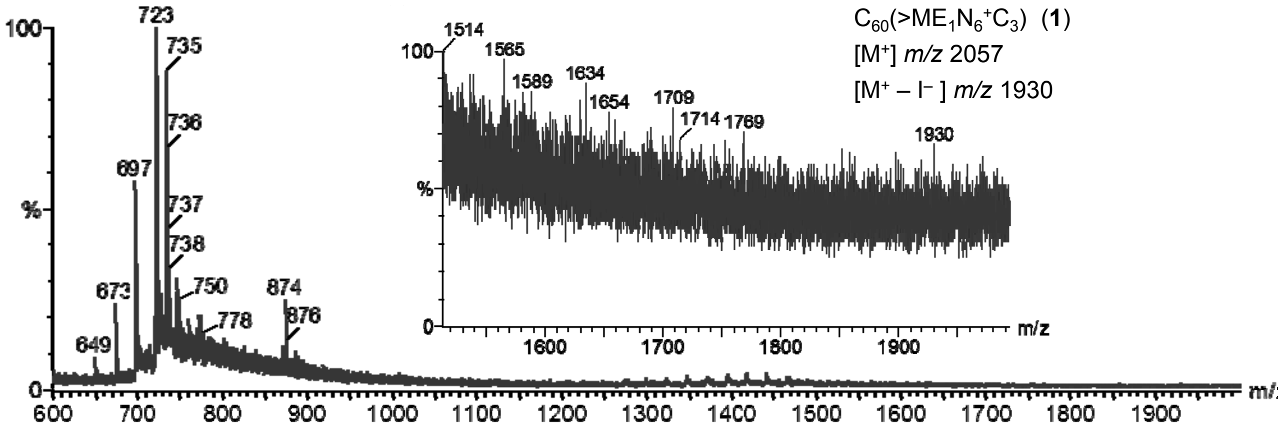

Supplement: Supplementary file 1 [file molecules-17-05225-s001.pdf]
